# New-Onset Myocarditis in an Immunocompetent Adult with Acute Metapneumovirus Infection

**DOI:** 10.1155/2015/814269

**Published:** 2015-09-02

**Authors:** Mark A. Weinreich, Ahmad Y. Jabbar, Nagina Malguria, Robert W. Haley

**Affiliations:** ^1^Parkland Health and Hospital Systems and Department of Internal Medicine, University of Texas Southwestern Medical Center, Dallas, TX 75390-8874, USA; ^2^Parkland Health and Hospital Systems and Department of Radiology, University of Texas Southwestern Medical Center, Dallas, TX 75235, USA

## Abstract

*Introduction*. A number of viruses have been implicated in viral myocarditis; however, there has been no previous report of human metapneumovirus (hMPV) causing this condition. Discovered in 2001, hMPV is typically associated with upper respiratory illness, mainly affecting children. *Case Presentation*. We report the case of a 25-year-old man with acute systolic heart failure from viral myocarditis secondary to the hMPV. The patient was initially admitted to the general medical ward but developed increasing oxygen requirements resulting in transfer to the cardiac intensive care unit. Cardiac magnetic resonance imaging was used to help confirm the diagnosis. He was treated with intravenous diuretics, and afterload and preload agents, and he was subsequently discharged home after seven days of hospitalization. *Discussion*. hMPV is typically a respiratory pathogen; however, it was associated with in myocarditis in our patient. Due to the recent ability to detect this virus, we may see more cases of this, particularly during peak months of infection. *Conclusion*. This is the first case description of myocarditis associated with hMPV infection.

## 1. Introduction

Myocarditis of infectious etiology is most often due to viruses, of which over 20 viruses have been identified as causative agents. Adenovirus, enterovirus, and parvovirus B19 are among the most common viruses associated with myocarditis [1]. The incidence of viral myocarditis is unknown because the diagnosis typically requires endomyocardial biopsy since there is no noninvasive gold standard for diagnosis. Inflammation from direct viral injury or a viral-induced autoimmune response is thought to be the mechanism resulting in myocarditis [2]. Patient presentation can range from fulminant, acute, or chronic heart failure. Diagnosis is based on classic clinical history, supportive laboratory tests, and cardiac imaging [3].

The human metapneumovirus (hMPV) has never previously been described as a cause of viral myocarditis. First discovered in 2001, it has a seasonal variation with outbreaks occurring in the northern hemisphere during January to March [4, 5]. It primarily affects children and has been detected in 4–16% of patients with acute respiratory tract infections [5]. It can affect adults of all ages, however, with a wide spectrum of respiratory diseases including severe respiratory failure [6]. Common symptoms include fever, cough, rhinorrhea, and dyspnea [7, 8]. Currently, no evidence exists to determine if hMPV is limited to the respiratory tract or can affect different organ systems [5]. There is some evidence, however, of its presence in middle ear fluid and in brain tissue [9, 10]. Our report would be the first to describe its affecting the heart.

## 2. Case Presentation

**Figure 1:**
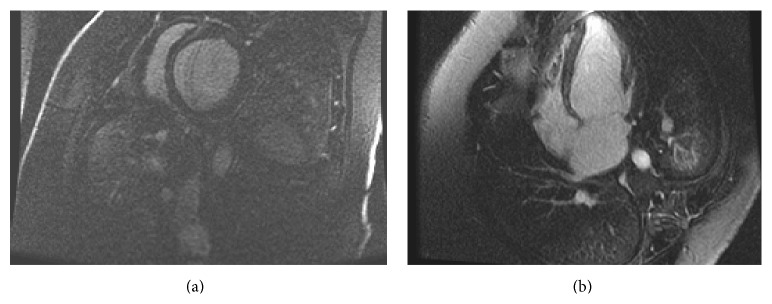
Short axis (a) and four-chamber (b) delayed hyperenhancement images demonstrate midwall septal enhancement of the left ventricle, suggestive of myocarditis in this clinical context.

A 25-year-old man with no significant past medical history presented with persistent fever and nonproductive cough of two-week duration. His symptoms were initially treated as an outpatient one week prior to presentation with amoxicillin/clavulanic acid due to concern for pneumonia; however, his symptoms progressively worsened. Upon presentation, his initial vital signs were as follows: T: 100.8, P: 108, BP: 120/73, RR: 20, and oxygen saturation: 95% on room air. Within 24 hours he developed increased oxygen requirements requiring three liters of supplemental oxygen, orthopnea, and voluminous blood-tinged, frothy sputum. Physical examination was remarkable for mild respiratory distress and diminished heart and lung sounds. Initial basic metabolic panel and complete blood count were normal. NTproBNP was elevated at 513, troponin-t was elevated at 0.04, and inflammatory markers were elevated (ESR 55, CRP 13.4). Respiratory viral reverse transcriptase polymerase chain reaction (PCR) was positive only for hMPV using a nasopharyngeal swab. Chest X-ray showed new cardiomegaly and bilateral opacities, as compared to a normal chest X-ray 6 months earlier, suggesting pulmonary edema. Our differential diagnosis at this point included acute heart failure, pulmonary infection, and diffuse alveolar haemorrhage. Pulmonary medicine was consulted and a computed tomography (CT) of the chest demonstrated bilateral opacities. Echocardiogram demonstrated a severely depressed ejection fraction of 25% with severely enlarged left atrial volume and severely dilated left ventricle. Cardiac magnetic resonance imaging (MRI) showed septal wall enhancement compatible with myocarditis (Figure 1). The patient was transferred to the cardiac intensive care unit and started on intravenous diuretics and afterload and preload reducing agents (furosemide 80 mg IV BID, captopril 3.125 mg TID, metoprolol tartrate 12.5 mg BID, and isosorbide dinitrate 10 mg TID). Over the course of seven days the patient improved. He was net negative eight liters from admission and was discharged home on lisinopril 5 mg and metoprolol succinate 25 mg daily.

On hospital day 4, the national reference laboratory's report of respiratory pathogen testing of a nasal swab collected on admission was positive for hMPV but negative for pathogens most commonly causing pulmonary infection, including influenza A and influenza B, parainfluenza, respiratory syncytial virus, adenovirus, tuberculosis, and mycoplasma.

## 3. Discussion

This is the first case description of viral myocarditis associated with human metapneumovirus. The void in literature of hMPV as a cause of viral myocarditis may be due to the relative recent ability to detect this virus or possibly something intrinsic to the virus. There is emerging evidence that hMPV may not be limited to the respiratory tract, and our report would support this [9, 10].

Our patient presented with acute onset of severe systolic dysfunction in the setting of a two-week history of upper respiratory illness, which is consistent with acute viral myocarditis. We investigated for various infectious etiologies of his myocarditis and he tested positive only for hMPV. His clinical history and viral prodrome fit with this as the etiology and were not compatible with other viral etiologies of myocarditis including cytomegalovirus and Epstein-Barr virus. Past reports indicate that myocarditis presenting in patients with new-onset heart failure and an influenza-like illness testing positive for a single viral pathogen only is typically shown to be viral myocarditis [11–13]. We made the diagnosis of viral myocarditis by the clinical history, elevated inflammatory markers and cardiac enzymes, echocardiogram with severely reduced ejection fraction, and a cardiac MRI suggestive of myocarditis. Cardiac MRI often shows signs typical of myocarditis and is becoming routine in diagnosis [14]. The diagnosis on cardiac MRI is based on detection of edema on T2 weighted images and patchy or diffuse enhancement in midwall and/or subepicardial areas of the left ventricle [15]. While this pattern of enhancement may be seen in other nonischemic cardiomyopathies, particularly sarcoidosis, idiopathic dilated cardiomyopathy, or Anderson Fabry disease, the acuity of clinical presentation and correlation with other clinical features of myocarditis differentiate myocarditis from these conditions. In addition, fibrosis on cardiac MRI has prognostic significance [16]. One study found that edema visualized on cardiac MRI is associated with positive viral genome detection by PCR; however, this feature was not present in our case [17]. The patient's echocardiogram showed dilated left ventricle and atrium which is frequently seen in acute myocarditis where they indicate a worse prognosis [18, 19]. We did not perform an endomyocardial biopsy since it would not have changed the patient's management. Whereas the definitive diagnosis of viral myocarditis does require endomyocardial biopsy, its utility has been called into question, and there are now specific indications for this procedure [20, 21].

The patient was treated with intravenous diuretics, an ACE-inhibitor, and a beta blocker. Published recommendations for viral myocarditis suggest only symptomatic treatment with medications added as indicated by the patient's New York Heart Association functional class [22]. Specific treatment options for viral myocarditis have not been established. Immunosuppressive and immune modulating agents do not appear to be effective, and the benefit of antiviral medications is unclear [23].

Our patient was discharged home after a seven-day hospital stay. The prognosis of viral myocarditis is variable. Patients with fulminant myocarditis tend to have an initial increase in mortality but excellent long term survival, whereas patients with acute myocarditis tend to be less ill initially but have worse long term outcomes [22]. Other studies have shown that patients with myocarditis and an ejection fraction of <45%, as in the reported patient, tend to have worse outcomes [24].

## 4. Conclusions

This is the first case report of myocarditis caused by hMPV. hMPV is well known to cause respiratory infections, but emerging evidence has suggested it can affect other organ systems. This case report would support this. Due to the relatively recent ability to detect this virus, we may see more cases of this, specifically during its peak months of infection. Severe infections associated with this virus are typically seen in children, elderly, and immunocompromised patients; however, our patient was a young adult. He had new onset of a severely depressed ejection fraction and was transferred to the cardiac intensive care unit. He responded well to intravenous diuretics and afterload and preload reducing agents and was discharged home after seven days of hospitalization.
